# Viral surface geometry shapes influenza and coronavirus spike evolution through antibody pressure

**DOI:** 10.1371/journal.pcbi.1009664

**Published:** 2021-12-13

**Authors:** Assaf Amitai

**Affiliations:** 1 Institute for Medical Engineering and Science, Massachusetts Institute of Technology, Cambridge, Massachusetts, United States of America; 2 Ragon Institute of MGH, MIT, and Harvard, Cambridge, Massachusetts, United States of America; 3 Department of Chemical Engineering, Massachusetts Institute of Technology, Cambridge, Massachusetts, United States of America; Imperial College London, UNITED KINGDOM

## Abstract

The evolution of circulating viruses is shaped by their need to evade antibody response, which mainly targets the viral spike. Because of the high density of spikes on the viral surface, not all antigenic sites are targeted equally by antibodies. We offer here a geometry-based approach to predict and rank the probability of surface residues of SARS spike (S protein) and influenza H1N1 spike (hemagglutinin) to acquire antibody-escaping mutations utilizing in-silico models of viral structure. We used coarse-grained MD simulations to estimate the on-rate (targeting) of an antibody model to surface residues of the spike protein. Analyzing publicly available sequences, we found that spike surface sequence diversity of the pre-pandemic seasonal influenza H1N1 and the sarbecovirus subgenus highly correlates with our model prediction of antibody targeting. In particular, we identified an antibody-targeting gradient, which matches a mutability gradient along the main axis of the spike. This identifies the role of viral surface geometry in shaping the evolution of circulating viruses. For the 2009 H1N1 and SARS-CoV-2 pandemics, a mutability gradient along the main axis of the spike was not observed. Our model further allowed us to identify key residues of the SARS-CoV-2 spike at which antibody escape mutations have now occurred. Therefore, it can inform of the likely functional role of observed mutations and predict at which residues antibody-escaping mutation might arise.

## Author summary

The immune system responds to viruses by making neutralizing antibodies to regions of the viral spike protein, which mutates to escape. To inform vaccine design and understand how the fitness landscape of the viral spike changes over time, it is necessary to identify and quantify the factors directing its evolution. Based on the 3D structure of the viral surface and spike as captured with Cryo-EM and crystallography, we aimed to create a coarse-grained model for the effect of antibodies in forcing surface residues of the spike to mutate. We found that for pre-pandemic influenza (hemagglutinin) and the corona sarbecovirus subgenus (S protein), the location of a residue on the spike protein, which modulates its accessibility to antibodies, highly correlates with its propensity to mutate. Hence, a mechanistic approach can be used to identify aspects of viral spike sequence diversity related to antibody escape.

## Introduction

The COVID-19 pandemic, caused by the SARS-CoV-2 coronavirus, is one of the most challenging global health crises of the century [1]. The virus emerged as a result of a zoonotic shift [2,3]. It is a member of the betacoronaviruses family [4], related to coronaviruses found in bats [5], and to SARS CoV which causes severe respiratory syndrome [6].

Coronaviruses (CoVs) have the largest genomes among RNA viruses [7]. Nonstructural protein 14 (nsp14), a subunit of the replicase polyprotein encoded by CoVs is thought to provide a form of proofreading activity, which could support the expansion of large CoVs genomes to their current size. One result of such proofreading activity is that CoVs genomes are less mutable compared to other RNA viruses [8], and thus the sequence diversity of SARS-CoV-2 is quite low [9].

In response to the SARS-CoV-2 pandemic, many approaches for antibody (Ab) therapies, and vaccines have been explored [10]. Almost all vaccination approaches aimed to use the glycoproteins or spike protein (S) of the virus in its trimeric form [11] or vaccinate with the full (inactivated) virus [12]. The spike, a class I fusion glycoprotein, mediates entry to the host cell by binding to the angiotensin-converting enzyme 2 (ACE2) receptor [4] and is the main target of Ab response [13]. These therapeutic approaches have been successful in eliciting strong Ab and T cell response against the virus [14] and in particular, Abs against the receptor-binding domain (RBD) of the spike, which have been shown to have neutralization and protective capabilities [13, 15].

Since its zoonotic shift, SARS-CoV-2 acquired several key mutations. One mutation at the spike (D614G) is now widespread and is thought to support a high viral growth rate [16]. Others, such as N501Y and E484K are associated with escape from Ab response [17]. Ab escape is common in other RNA viruses such as the influenza virus, which causes seasonal epidemics and occasional pandemics. A major pandemic event occurred in 2009 when the H1N1 influenza A virus performed a zoonotic shift from swine to humans [18]. To evade immune memory, influenza spike, hemagglutinin (HA), rapidly acquires mutations from one year to the next [19,20].

Given the prevalence of these viruses, to inform vaccine design and understand how the fitness landscape of the viral spike evolves, it is important to recognize residues where mutations would permit the virus to escape Ab pressure and evade immune protection, secondary to natural infection or vaccination efforts.

Here we sought to understand and predict the extent to which the mutations at the spikes of influenza and the sub-family of SARS-CoV-2 could be attributed to Ab pressure. The magnitude (titers) of Ab response against a given epitope is a direct consequence of the B immunodominance hierarchy patterns of an immunogen, which are the result of various aspects of the humoral response to antigen [21–23]. Amongst them is the B cell repertoire–the number of B cell clones targeting different epitopes [24–29], their germline affinity [24,30], and T cell help to B cell [31]. In this study, we concentrate on the geometric presentation of the spike to Abs. We have previously shown, using coarse-grained molecular dynamics simulations, that the geometry of the immunogen spike presentation on the virus recapitulates the known immunodominance of the HA head compared to its stem [24].

**Fig 1:**
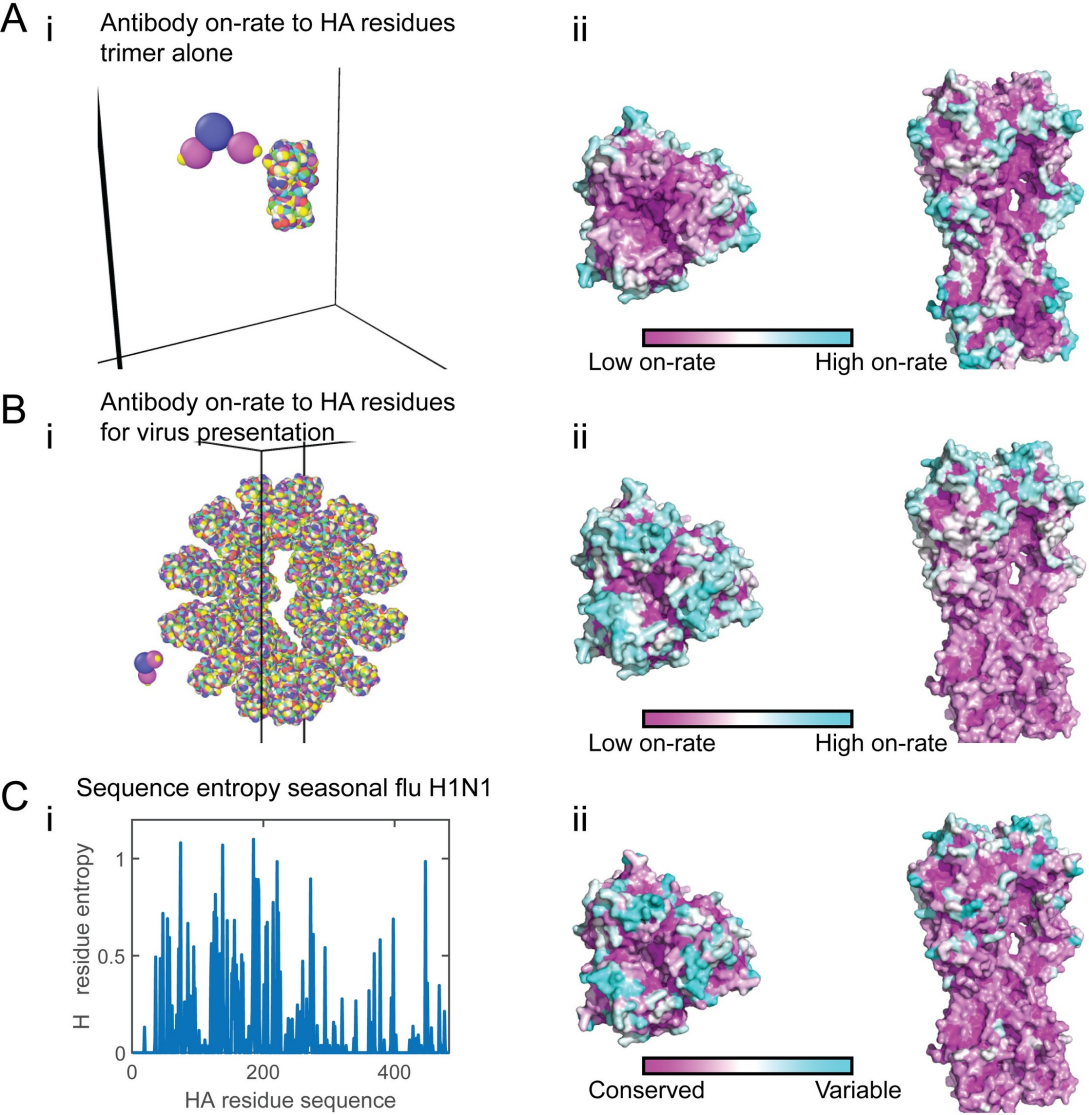
Antibody targeting and mutability of the hemagglutinin protein for the seasonal flu. **(A-B)** Coarse-grain model of the hemagglutinin trimer of A/New Caledonia/20/1999 (NC99) H1N1 influenza protein in its closed form (A). The virus model has 40 HA molecules at a spacing of 14.8 nm. [Measured spike spacing on influenza is 14 nm [78]] (B). For each immunogen geometry (trimer—A or full virus—B), a detailed atomistic structure of the immunogen is coarse-grained and presented in rainbow colors (panel i). Here every colored bead on the immunogen is a residue, representing a different HA epitope (228 different possible sites on trimeric HA). The antibody structure is presented as the Fc (blue bead), two arm (magenta beads) and antigen binding fragment (Fab) (yellow beads). Panels ii within A-B depict coarse-grained simulations for the on-rate of the Ab first arm binding (see Eq (S4)) to these residues [data from [24]]. The on-rates estimated from the simulation are superimposed on the HA structure. Top view (left), side view (right). The on-rate to cyan residues is high, intermediate to white residues, low for purple residues, and was the average of multiple simulations. **(C)** Panel i depicts the entropy (see Eq (1)) of HA epitopes computed for the seasonal flu (pre-pandemic influenza H1N1 (1918–1957 and 1977–2009) (sequences from [40]). Panel ii shows the entropy of the residues superimposed on HA structure, where highly mutable residues are in cyan, intermediate in white and conserved residues in purple.

We developed an in-silico approach to estimate the IgG Ab targeting—a proxy for B cell immunogenicity [24] of residues on the spike surface, and the differential accessibility to antigenic epitopes due to the geometrical presentation of spikes on the surface of the virus. To this end, we computed the on-rates of a coarse-grained Ab model to surface residues on the spike (Fig 1A and 1B). Superimposing these on-rates on the spike surface gives the *Ab on-rate maps* of influenza and corona spikes, which we applied to predict how the antigenic space is explored unevenly by Abs across the spike surface. We then used sequences from public repositories (www.ncbi.nlm.nih.gov, www.gisaid.org) to compute the sequence diversity of the same surface residues, utilizing Shannon’s entropy. Superimposing the entropy on the spike surface gives its *mutability map*. Next, we compared the on-rate and mutability maps and found a high degree of correlation between them. We found that about 50% of the mutability map variability of the S protein of the severe acute respiratory syndrome-related betacoronavirus (sarbecovirus), and 67% of the variability in the mutability map of the seasonal influenza spike (HA) can be attributed to the uneven accessibility of surface residues by Abs (antibody pressure). This high degree of correlation suggests that average, polyclonal Ab pressure modulated by spike presentation geometry on the viral surface was consequential in the diversification of the coronavirus sarbecovirus spike and the seasonal flu spike.

We further studied the time evolution of SARS-CoV-2 spike mutability up to May 31^st^, 2021. We found that the correlation between the spike mutability and our model gradually increased, suggesting diversification at residues that are highly targeted by Abs, before rapidly falling between January and May 2021. Finally, we found that several residues predicted by our model to be highly targeted by Abs have now acquired key mutations that are associated with Ab escape, validating our approach. Overall, our approach allows us to recognize, based on the 3D structure of glycoprotein and cryo-EM images of the viral surface, whether their mutational landscape has features suggesting Ab evasion, and rank surface residues according to their likelihood to acquire Ab-escaping mutations in the future. Importantly, this approach can detect signs of SARS-CoV-2 and influenza adaptation to evade immune pressure by memory B cells.

## Results

### Geometry-dependent on-rate of Abs to HA epitopes

**Fig 2:**
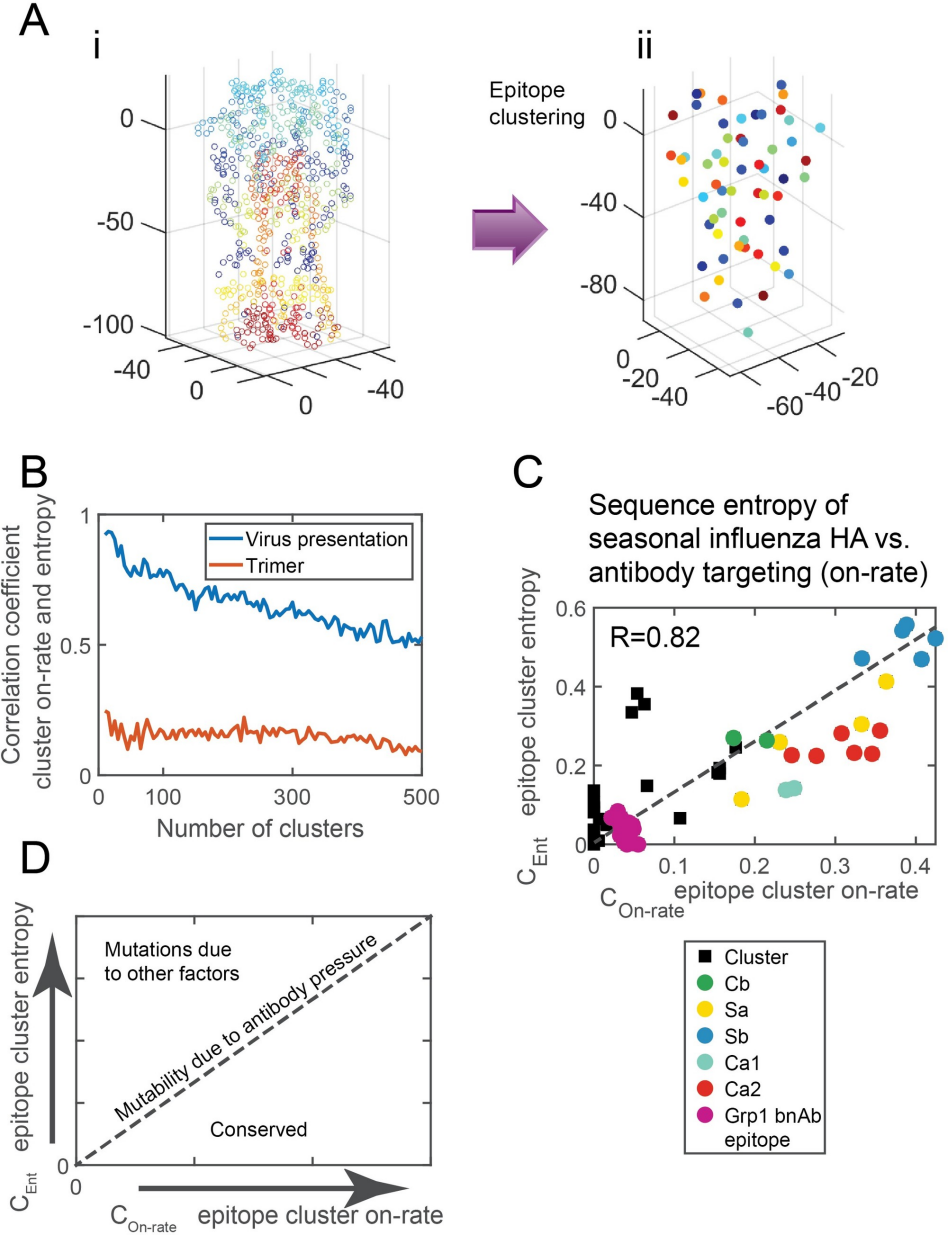
Antibody pressure guided the mutability of the hemagglutinin. **(A)** Panel i. HA protein. Each circle corresponds to a surface residue (epitope) and was colored differently for illustration. Panel ii. Surface residues (epitopes) were clustered (see Materials and Methods). Each epitope cluster is was colored differently for illustration. **(B)** The correlation coefficient between epitope cluster entropy (Eq (2)) and the epitope cluster on-rate (Eq (3)), as a function of cluster number, computed for HA in the virus presentation depicted in Fig 1B (blue), and at the trimer presentation depicted in Fig 1A (red). **(C)** Scatter plot of the epitope clusters entropy computed for the seasonal influenza H1N1 vs. the epitope clusters on-rate (the number of clusters is 60). The correlation coefficient between them is 0.82. Marked are clusters containing residues belonging to the five known antigenic sites of flu (Cb—green, Sa—yellow, Sb—blue, Ca1—cyan, Ca2—red). Also marked is the group 1 conserved broadly neutralizing antibodies epitope (purple). **(D)** Schematic of the relationship between entropy and computed Ab on-rate for circulating viruses evolving under Ab pressure.

The high-density presentation of spikes on the viral surface shelters, through steric impediments, immunologically recessive and conserved residues from Ab targeting–for example, residues belonging to the stem of HA [20,28,29]. To study the relative accessibility of residues on the spike surface, we employed coarse-grained MD simulations to define how the on-rate of an IgG Ab model could be modulated by the presentation of the spike. We first studied two geometrically distinct HA-presenting immunogens: 1) Presentation as soluble full-length HA trimer in its closed form [A/New Caledonia/20/1999 (NC99)] [25, 32–34] (Fig 1A-i); 2) HA presentation within H1N1 influenza A (NC99) virus model (Fig 1B-i) (See Materials and Methods). For each presentation form, we computed the on-rates for Abs engaging different surface epitopes. The on-rate is the inverse of the mean first passage time of a single Ab arm to its target epitope (See Materials and Methods).

We superimposed the on-rates of the first Ab arm on the HA structure to represent its *on-rate map* (Fig 1A-ii and 1B-ii) [data from [24]]. In the context of the free HA trimer presentation, we found that residues at convex sections on the spike surface were more accessible to Abs, resulting in a higher on-rates (Fig 1A-ii). In the context of virus HA presentation (Fig 1B-ii), similar behavior followed, and the density of spikes on the viral surface reduced the ability of Abs to penetrate and interact with epitopes on the lower part of the spike, resulting in an on-rate gradient of the Abs targeting residues along the main axis (Fig 1B-ii right). Hence, presentation on the virus surface, as occurs in vivo, leads to an immunodominance or Ab pressure (targeting) gradient along the main axis of HA.

### Antibody pressure directs the evolution of the seasonal flu

Viral infection elicits a humoral response and the production of Abs that target residues on the surface of spikes. For circulating viruses to propagate in a population, they have to evade neutralization and recognition by Abs [35,36]. To do so, they accumulate mutations on their surface proteins [37,38]. Because sterically hidden residues are less accessible to Abs, we hypothesized their need to mutate is smaller compared to more accessible ones. Hence, we expected spike evolution and the mutational landscape to follow Ab pressure.

The influenza virus mutates from one year to the next, where most of the mutations are concentrated in five antigenic sites (Sa, Sb, Ca1, Ca2, and Cb) located at the head of HA [39]. Escape from neutralization by Abs is one of the main factors contributing to HA mutability [19,20]. To examine the relationship between Ab pressure and HA surface mutability, we studied the evolution of the pre-pandemic seasonal influenza virus H1N1 using sequences dating back to 1918 (see Materials and Methods). Following sequence alignment, we computed the entropy of each surface residue identified as an epitope (see Materials and Methods). The entropy of residue *j* is given by

$$H_{j}=\sum_{i\in [\mathrm{Amino}\;\mathrm{acids}]}p_{j,i}\log(p_{j,i}),$$ (1)

where *p*_*j*,*i*_ is the probability of amino acid *i* to appear at residue *j* across the viral population (Fig 1C-i). By superimposing the residues entropy on the surface of HA, we created its *mutability map* (Fig 1C-ii). Interestingly, the mutability map is comparable to the on-rate map for the virus presentation (Fig 1B-ii), showing a pronounced pattern of diminishing mutability gradient along the main axis of the spike. This is corroborated by previous studies showing that the HA head acquires more mutations and evolves faster than its lower part–the stem [40].

To quantify the similarity of the on-rate and mutability maps, we aggregated close-by residues on the spike surface into clusters of size *k* (Fig 2A) (See Materials and Methods, and S2 Fig), and computed for each epitope cluster *k* its entropy and on-rate as follows:

$$C_{Ent,k}=\frac{1}{N_{k}}\sum_{j\in [\mathrm{Resiudes}\;\mathrm{in}\;\mathrm{cluster}\;k]}H_{j},$$ (2)

$$C_{On-rate,k}=\frac{1}{N_{k}}\sum_{j\in [\mathrm{Resiudes}\;\mathrm{in}\;\mathrm{cluster}\;k]}\omega_{j},$$ (3)

where *C*_*Ent*,*k*_ and *C*_*On−rate*,*k*_ are the epitope cluster entropy and epitope cluster on-rate respectively, *N*_*k*_ is the number of residues in cluster *k*, *ω*_*j*_ is the on-rate of the first Ab arm to residue *j*, and the entropy *H*_*j*_ of residue *j* is given by Eq (1).

To assess the predictive strength of the on-rate map in explaining the observed mutability, we computed the correlation between *C*_*On−rate*,*k*_ and *C*_*Ent*,*k*_ as a function of the cluster number (Fig 2B). We find that the correlation values for HA virus presentation are high, with a maximum of 0.92 for 10 clusters. Intriguingly, the correlation value, regardless of cluster number, is always larger for the virus presentation than for the trimer (Fig 2B), highlighting that spike evolution and escape due to Ab pressure occurs in the context of the virus—as a result, both mutability and Ab on-rate vary most along the main axis of the spike. We determined the optimal number of clusters to be *k* = 60 (See Materials and Methods). For *k* = 60, we found a correlation of 0.82 between *C*_*Ent*,*k*_ and *C*_*On−rate*,*k*_, suggesting that epitope cluster on-rate map, at this resolution, could explain 67% of the variability in the mutability map of HA.

Surprisingly, most epitope clusters that contain residues belonging to the five antigenic sites show a linear relation between their entropy and on-rate, suggesting that the mutability of these sites follows from their position on HA, the geometry of its presentation on the viral surface, and is due to Ab pressure (Fig 2C). Epitope clusters containing conserved residues at the HA stem belonging to the HA Group 1 broadly neutralizing epitope [20,28,29] similarly align.

Taken together, these results suggest that the mutability of surface spike epitopes of circulating viruses can be roughly described using a diagram (Fig 2D). The mutability of epitope clusters that lay on a linear line of epitope cluster entropy vs. epitope cluster on-rate is related to the average Ab pressure acting on these residues (Fig 2D). Epitope clusters below the line are more conserved than would be expected based on their accessibility to Ab pressure and this could be due to the presence of functionally important sites. Epitope clusters above that line are more mutable than would be expected due to Ab pressure and may result from allosteric immune escape [41], escapes from CD8+ T cells [42,43], glycosylation [44], or other factors.

### The mutability map of the sarbecovirus spike follows geometry-dependent antibody pressure

**Fig 3:**
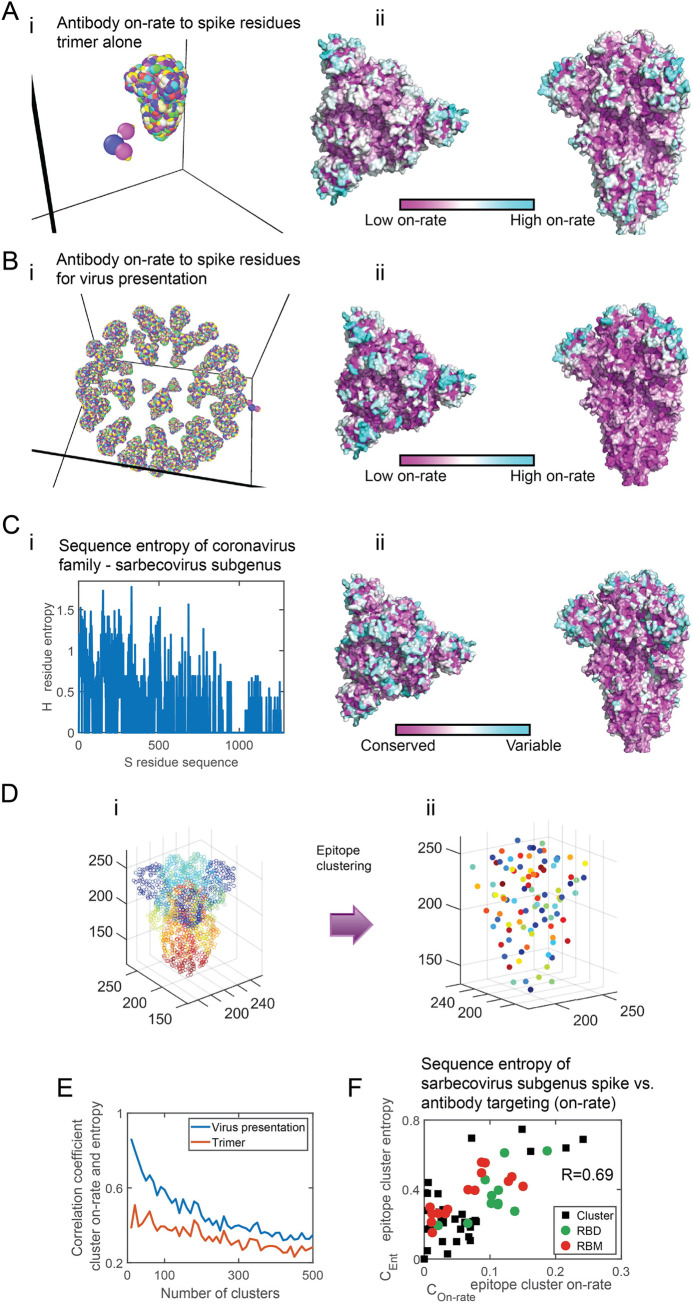
Antibody targeting and mutability of the sarbecovirus subgenus spike. **(A-B)** Coarse-grain model of the SARS-CoV-2 spike (S protein) in its closed form (A) [45]. (B) The virus model has 65 S molecules at a density of 0.27 spikes per 100nm^2^ [46]. A detailed atomistic structure of the spike is coarse-grained and presented in rainbow colors (panels i). Every colored bead on the spike is a residue, representing a different S epitope (255 different possible sites on trimeric S). Panels ii depict coarse-grained simulations for the Ab on-rate to these residues (see Fig 1A-ii for definition and color-coding). **(C)** Panel i depicts the entropy (see Eq (1)) of each spike residue computed for the sarbecovirus subgenus spike (see Table 1). Panel ii shows the entropy of the residues superimposed on the spike structure. Same color-coding as in Fig 1C-ii. **(D)** Panel i. The spike protein of the coronavirus. Each circle corresponds to a surface residue (epitope) and was colored differently for illustration. Panel ii. Surface residues (epitopes) were clustered (see Materials and Methods). Each epitope cluster is was colored differently for illustration. The number of clusters is 60. **(E)** The correlation coefficient between epitope cluster entropy (Eq (2)) and the epitope cluster on-rate (Eq (3)), as a function of cluster number, computed for the corona spike in the virus presentation (blue), and at the trimer presentation (red). **(F)** Scatter plot of the epitope clusters entropy, computed for the sarbecovirus spike vs. the epitope cluster on-rate estimated from the simulations. The correlation coefficient between them is 0.69. Clusters that contain residues belonging to the RBD are in green and those containing residues belonging to the RBM are in red. (The number of clusters is 60).

To understand whether the geometrical principles controlling the distribution of mutation on the spike surface are general across species, we applied our computational model to study the mutability of the spike protein of close relatives of SARS-CoV-2 –the sarbecovirus subgenus. We considered two presentations of the corona spike (S protein) to Abs: 1) Presentation as soluble full-length S trimer in its closed form [45] (Fig 3A-i); 2) S presentation on the coronavirus surface (Fig 3B-i) [based on the cryo-EM structure of SARS that has 65 spikes on its surface [46] and SARS-CoV2 spike [45] (See Materials and Methods)].

We first computed the Ab on-rate to surface residues of S, when presented as a trimer or the surface of the virus model (Fig 3A-i and 3B-i, and Materials and Methods). Similar to our observation for the Abs on-rate against HA, we found an increased on-rate to convex regions, an on-rate gradient along the main axis of S for the virus presentation (Fig 3B-ii), but not for the trimer presentation (Fig 3A-ii). Next, we analyzed sequences of close relatives of the SARS-CoV-2 spike within the sarbecovirus subgenus (Table 1). Following alignment and construction of the phylogenetic tree (S3 Fig), we computed the mutational entropy of each surface residue identified as an epitope using Eq (1) (Fig 3C-i) and superimposed it on the spike surface to create its mutability map (Fig 3C-ii). We observed that the most significant change in mutability is along the main axis of S. To compare the on-rate and mutability maps, we applied the diffusion map transformation on (S2B Fig) and clustered the epitopes (Fig 3D). Studying the correlation value as a function of cluster size (Fig 3E), we found that the correlation between the on-rate and the mutability maps is always higher for the virus spike presentation compared to the trimer, highlighting that the geometrical context in which Abs interact with the spike determines its mutability. We found a high degree of correlation (R = 0.69) between *C*_*Ent*,*k*_ and *C*_*On−rate*,*k*_, suggesting that on-rate as computed by our model, at this resolution, can explain 48% of the variability in the mutability map of S (Fig 3F). The high degree of correlation between the maps suggests that average Ab pressure shaped, to the first order, the mutability of the sarbecovirus subgenus spike. While for seasonal influenza, the HA entropy (Fig 1C) was the result of a gradual accumulation of mutations over time, S protein entropy (Fig 3C) analyzed here is the result of a horizontal mutational process occurring simultaneously in different hosts, suggesting the virus evolves under similar geometrical immunoglobulin pressure.

**Table 1:** Sarbecovirus. Species used for the analysis detailed in Fig 3C.

Coronavirus Species	Collection date	Reference	Isolation origin
SARS-CoV-2 consensus	2019–2020	Computed in this manuscript	Human
Bat coronavirus RaTG13	2013	https://www.ncbi.nlm.nih.gov/protein/QHR63300.2	Rhinolophus affinis
Bat coronavirus Urbani	May 2003	https://www.ncbi.nlm.nih.gov/protein/AAP13441	Human
Bat coronavirus CUHK-W1	2003	https://www.ncbi.nlm.nih.gov/protein/AAP13567.1	Human
Bat coronavirus GZ02	2003	https://www.ncbi.nlm.nih.gov/protein/AAS00003	Human
Bat coronavirus A031	2004	https://www.ncbi.nlm.nih.gov/protein/AAV97988.1	Raccoon dogs
Bat coronavirus A022	2004	https://www.ncbi.nlm.nih.gov/protein/AAV98003.1	Raccoon dogs
Bat SARS-like ZXC21	2015	https://www.ncbi.nlm.nih.gov/protein/AVP78042.1	Rhinolophus sinicus
Bat SARS-like ZC45	2017	https://www.ncbi.nlm.nih.gov/protein/AVP78031.1	Rhinolophus sinicus
Bat SARS-like CoV Rp3/2004	2004	https://www.ncbi.nlm.nih.gov/protein/AAZ67052.1	Rhinolophus ferrumequinum
SARS coronavirus Rs 672/2006	2006	https://www.ncbi.nlm.nih.gov/protein/ACU31032.1	Rhinolophus sinicus
Bat SARS-like coronavirus WIV1	2012	https://www.ncbi.nlm.nih.gov/protein/AGZ48828.1	Rhinolophus sinicus
SARS-like coronavirus WIV16	2013	https://www.ncbi.nlm.nih.gov/protein/ALK02457.1	Rhinolophus sinicus

The receptor-binding domain (RBD) is involved in the spike binding to ACE-2 [47,48]. It has been shown that neutralizing Abs targeting the RBD can offer protection [15]. Within the RBD, residues belonging to the receptor-binding motif (RBM) are most important in binding to ACE-2. We recognized epitope clusters to which residues part of the RBD and RBM belong (Fig 3F). Many of the epitope clusters have both high entropy and high on-rate, which could suggest mutations acquired at these key domains across the spike are due to evasion from Abs, as well as adaptation to the host-specific receptor. Several of the highly targeted and mutable epitope clusters are not part of the RBD. Hence, Abs targeting these residues will not necessarily offer neutralization activity. However, Abs targeting these clusters can control viral infection through non-neutralizing pathways [49], thereby motivating the virus to mutate these highly targeted parts.

### SARS-CoV2 and the 2009 influenza pandemic spikes mutability is not predominantly due to antibody-pressure

**Fig 4:**
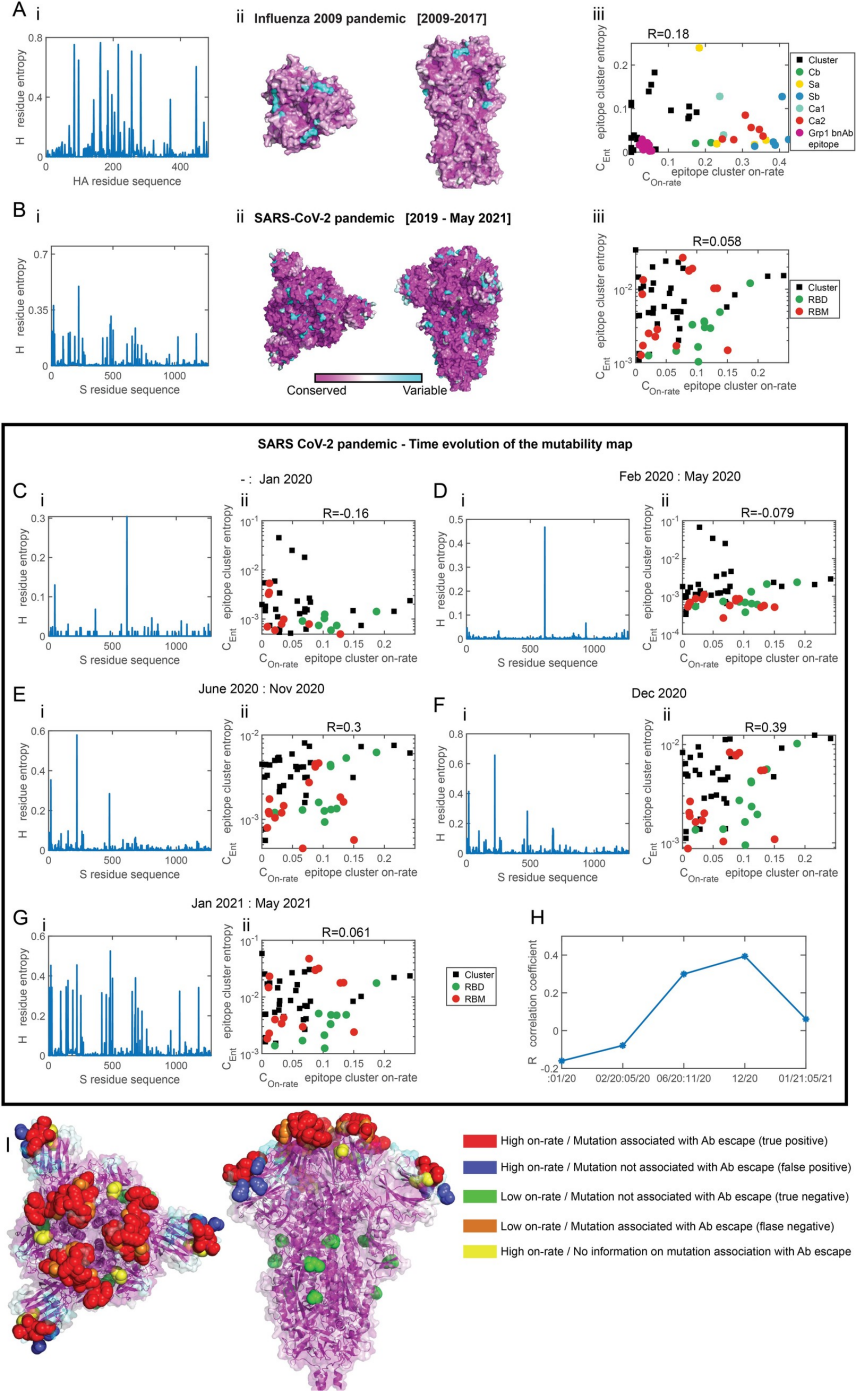
Spike evolution of the 2009 influenza pandemic and SARS-CoV-2. Comparison of the diversity of sequences (mutability map) and the on-rate maps. For A-B, panel i depicts the residue entropy as a function at different positions. For A-B, panel ii depicts the entropy of the residues is superimposed on the spike. Same color coding as in Fig 1C-ii. Panel iii. Scatter plot of the entropy of epitope clusters, against the epitope cluster on-rate computed for the spike. **(A)** Sequence entropy of HA for the pandemic flu H1N1 (2009–2017) (sequences taken from www.gisaid.org, and [40]). The correlation coefficient between the epitope cluster entropy and on-rate is R = 0.18. **(B)** Sequence entropy of the S spike protein of SARS-CoV-2 computed for all S protein sequences up to May 31^st^ 2021 (sequences downloaded from www.gisaid.org). The correlation coefficient between the epitope cluster entropy and epitope cluster on-rate is R = 0.058. Same legend as Fig 3F. **Time-dependence sequence entropy of SARS-CoV-2.** The entropy of the S spike protein of SARS-CoV-2 computed for sequences collected at 5 time periods since the beginning of the pandemic (panel i) and correlation to the on-rate map, following epitope clustering (panel ii) (same clusters as those shown and used in Fig 3D-ii and 3F). **(C)** up to February 1st 2020, R = -0.16, **(D)** February-May 2020, R = -0.079 **(E)** June-November 2020, R = 0.3, **(F)** December 2020, R = 0.39, **(G)** January-May 2021, R = 0.061. **(H)** The correlation coefficient as a function of time. (Find an interactive, comparison of the time-dependent mutability map to the on-rate map here https://amitaiassaf.github.io/SpikeGeometry/SARSCoV2EvoT.html). **Functional role of SARS-CoV-2 spike mutations. (I)** Residues where key mutations were identified in SARS-CoV-2 variants are marked with colored beads. Residues were ranked based on their on-rate (targeting) by Abs according to the model prediction. The upper 66^th^ on-rate percentile rank is the threshold between “high on-rate” (mutation due to escape) and “low on-rate” (mutation due to other factors) residues. Red residues have high on-rate and mutations in them were found to confer Ab escape (true positives) [Residue positions: 136, 140, 141, 143, 244, 345, 441, 444, 447, 449, 450, 452, 489, 490 493, 499, and 501]. Blue residues have a high on-rate and mutations in them have not been shown as of yet to not confer Ab escape (false positives) [Residues: 69, 80, and 138]. Green residues have a low on-rate and mutations in them do not confer Ab escape (true negatives) [Residues: 614, 655, and 701]. Orange residues have a low on-rate and mutations in them confer Ab escape (false negatives) [Residues: 346, 439, and 453]. Yellow residues have a high on-rate but it is unknown whether mutations in them confer Ab escape [Residues: 102, 367]. See S1 Data for a complete list.

Our analysis suggested that Ab pressure imposed by spike presentation geometry highly correlated with the mutational entropy of viruses circulating either over long periods (influenza) or across species (sarbecoviruses). To determine whether this observation can be generalized to pandemics, we computed the sequence entropy of HA for the 2009 flu pandemic H1N1 (sequences from [40], GISAID) (Fig 4A-i). Superimposing the entropy on the HA structure (Fig 4A-ii), we did not observe immunodominance gradient along the main axis of HA observed in the computational model (Fig 1B-ii). Unlike for the mutability of seasonal flu, the correlation coefficient between epitope cluster pandemic entropy and epitope cluster on-rate was low (0.18) (Fig 4A-iii).

SARS-CoV-2 zoonotically shifted to humans in 2019 [5], probably from bats via pangolins, although its precise evolutionary path is still unclear. Since then, it has spread in the human population, infecting more than 181 million people as of June 2020. To analyze its total mutational entropy up to May 31^st,^ 2021, we downloaded publicly available SARS-CoV-2 sequences from GISAID (www.gisaid.org) [50] (sequences choice is discussed in Materials and Methods), computed the sequence entropy (Fig 4B-i) and the mutability map (Fig 4B-ii). Interestingly, the mutability map does not show the same gradient pattern as observed for the sarbecovirus subgenus spike entropy (Fig 3C-ii). We applied the same clustering (*k* = 60) to compare the epitope cluster entropy and on-rate and found a low value of the correlation coefficient (0.058) (Fig 4B). Hence, we suggest that the total sequence entropy of SARS-CoV-2 thus far is not dominated by escape from Ab mutations.

### Time evolution of SARS-CoV-2 mutability map

To see if we could observe changes in the evolution trend of the virus of the time, indicative of Ab escape mutations, we separated SARS-CoV-2 sequences into five groups based on the time at which they were captured: 1] before 02/2020, 2] 02/2020-05/2020, 3] 06/2020-11/2020, 4] 12/2020, and 5] 1/2021-05/2021 (Fig 4C, 4D, 4E, 4F and 4G). Computing the correlation coefficient between the on-rate and mutability maps, we found an increase over time (Fig 4H) from a value of -0.16 before Feb 2020 to a peak of 0.39 for sequences sampled during 12/2020. While the correlation value of 0.39 is still low, it suggests significant sequence diversity in circulating strains at residues that are highly targeted by Abs residues, in accordance with other reports [51]. Interestingly, between January and May 2021, the correlation value has been dropping. This could be due to the fixation of some of the escape mutations at the S protein RBD in dominant circulating strains. An increase over months and years in correlation value between the on-rate map computed by our model and the evolving mutational landscape of SARS-CoV-2 could indicate that its mutability patterns are being shaped by Ab pressure. (See time dependence here https://amitaiassaf.github.io/SpikeGeometry/SARSCoV2EvoT.html).

### Comparing model prediction with key mutations in SARS-CoV2

Finally, we directly compared the prediction of our model of the computed Ab on-rate as proxy for escape mutation with emerging SARS-CoV-2 variants. We ranked all the residues identified in our model according to the Ab on-rate toward them. According to the model, residues for which the on-rate is high (high rank) are more likely to mutate due to Ab escape compared to residues for which the on-rate is low (low rank), for which we predict mutations are likely to have a different functioncal role. Based on current knowledge about the association of key mutations with Ab escape (See S1 Data), we studied the predictive power of the model (Fig 4I). We defined residues that are in the top 66th percentile on-rate rank as likely to mutate to escape Abs. Hence, high rank residues in which escape mutations occurred are true positive predictions of the model. Amongst them are residues 501 (73rd rank percentile) where a dominant mutation (N501Y) is present in lineages (B.1.1.7, B.1.351, and P.1) and was found to reduce neutralization by Ab [52,53] and increase in affinity to ACE2 [54]. Highly ranked residues where mutations do not offer Ab escape are false positives–such as residue 69. Indeed, Δ69 has emerged independently in multiple strains but it is not reported to confer escape [55]. Low on-rate ranking residues in which mutations were found to offer fitness advantages not related to escape are true negatives. Amongst them is residue 614, which has a highly prevalent mutation (D614G) and is associated with increased infectivity and transmissibility but not with Ab escape [16]. Finally, residues such as 453 received a low rank but mutations in them (Y453F [54]) were found to offer escape are false negatives. Overall, the model informs of the likely functional role of observed mutations and predicts at which residues Ab-escape mutation might arise in the future.

## Discussion

Humoral immunity is often characterized by dominant versus recessive responses to different epitopes on the same antigen. This hierarchy of B cell immunodominance depends on many factors, amongst them are the precursor frequency within the germline B cell repertoire, B cell receptor affinity, and the steric accessibility or antigen geometry. Pathogens take advantage of antigen geometry to shield sites of vulnerability. Such is the case in the influenza spike hemagglutinin, where conserved sites are located in the sterically hidden stem [56–58], or on HIV spike gp120, where the vulnerable and evolutionary conserved CD4 binding site position does not allow Abs to form bivalent interactions, reducing Ab avidity [59]. While mature Abs are nevertheless capable of approaching sterically restricted sites via somatic hypermutations that could extend, for example, their CDR3 loops [60], immunogen shape and valency manipulates B cell immunodominance patterns, their selection process in the germinal center, or the expansion of memory B cell population [24,61,62]. Because viruses must evade Ab response to survive, B cells immunodominance patterns could roughly recapitulate spike mutability patterns. Immunogen shape that contributed to these patterns can be obtained directly from structural data and does not require prior knowledge about the immune repertoire. Thus, we studied whether spike presentation geometry to Abs is a good predictor of its mutability. Using a coarse-grained model of an Ab, HA, and the S protein of SARS-CoV-2, in both trimer and viral presentation model system, we computed the Ab on-rate maps as a proxy for Ab pressure on the spike. We used the on-rate maps to assess whether the magnitude of the mutability of surface residues is governed by geometrical considerations (Fig 2D).

We found that for the seasonal flu spike–HA, geometry through the presentation on the virus could explain, to the first order, the mutability pattern at its surface. In particular, the mutability of the five antigenic sites is ordered as would be expected by the geometric restriction imposed by their position on the spike, as did the conserved group 1 epitope, which is functionally important for HA conformation change (Fig 2C). Hence, we speculate that rather than maintaining functionally important sites conserved by negatively selecting mutants at such sites, the virus positions functional sites at a location, where their tolerable mutational rate would be determined by their need to escape from targeting by the average polyclonal Ab response.

To understand whether a similar principle governs the mutability of coronaviruses, we created a similar coarse-grained model of the SARS CoV family. As coronaviruses do not mutate much in comparison to other RNA viruses, we decided to analyze their mutability across the virus sub-species, using sequences isolated from different hosts in the years 2003–2019. In mammalians, these viruses have to evade immunoglobulin response which we hypothesized would lead to geometrically similar escape patterns. We found that geometry, through Ab targeting, shapes to the first order the mutability patterns on the sarbecovirus subgenus spike map. Hence, these viruses evolve across various hosts under roughly geometrically similar Ab pressure–at least the main axis of the virus seems to be the first principle axis of mutability, resulting from the density of spikes on the viral surface.

The mutational probability distribution we sampled for the sarbecovirus subgenus is analogous to sampling different “realizations” of the statistical ensemble of the sequence landscape of the viruses [63], where each realization is a virus from a different host. For the seasonal flu, we considered sequences over a long period—starting from 1918 and aggregated them to a single probability distribution analyzed. In both cases, presentation geometry roughly explained sequence entropy. Comparing both these approaches to describe mutability distribution is conceptually similar to the ergodic theorem in statistical physics, where the averages of a stochastic process sampled over time are equivalent to the averages computed over different statistical realizations. While evolution patterns of mutating viruses are not an ergodic system in general–as many mutants are not viable and hence unreachable in the sequence space, the similar geometry of immunoglobulins and spike presentations could be is the reason our model works for both these different instances, with mutations distributed across time (for influenza), or across species (for sarbecoviruses). Statistical physics models have been previously used [64] to analyze the sequence space to compute the fitness landscape space of viruses [65,66]. The overall fitness of viruses is often split into its intrinsic fitness of the virus and a fitness component related to evasion from the immune response (i.e. Abs) [67]. As our approach allows for a rough estimation of the virus Ab-dependent element of the fitness, it can be used as a prior in inference methods to extract the intrinsic fitness.

Because of its proofreading mechanism, SARS-CoV-2 is not expected to mutate much. Nevertheless, since the SARS-CoV-2 pandemic has erupted, its sequences have been analyzed to detect mutations that would increase its fitness, infection capabilities, or allow it to escape from Abs [16,51,53,68]. To determine patterns of escape due to Ab pressure on the SARS-CoV-2 spike, we compared its mutability map to the on-rate map over time and found an increase in the correlation value since the beginning of the pandemic up to December 2020, resulting from the larger sequence heterogeneity of S1 (Fig 4H). Between January and May 2021, we observed a drop in the correlation value. This could be due to the high prevalence of specific mutation (N501Y, Δ69–70, E484K, Y453F) [17] reducing S1 entropy while contributing to viral fitness through escape and increased binding at ACE2. It is also plausible that the vaccine, which was introduced around December 2020, where the spike is presented in a stable pre-fusion trimeric form [14], resulted in different patterns of Ab pressure than those elicited by infection. For the 2009 influenza pandemic, we similarly found a low correlation value of 0.18.

Pandemics are usually caused by a newly introduced pathogen to the human population that is poorly matched by the predominant immune responses and hence do not elicit strong immune memory. At the same time, a pandemic virus has not evolved to infect humans. Fitness advantage from positive mutations, shortly after the zoonotic shift, would likely result from increasing infectivity rather than allowing the virus to escape Abs. This would result in an initial low correlation between the mutability of pandemic influenza and SARS-CoV2 and the model. An example of such initial adaptation is the prevalent D614G mutation in the S protein of SARS-CoV2, which is associated with increased infectivity and transmissibility and not with escape [69].

Directly comparing our model for SARS-CoV2 with current knowledge on the effect of mutations, we found that our model successfully predicted the position of Ab-escape-related mutations in some of the widely circulating variants (B.1.1.298, B.1.1.7, B.1.351, and P.1) [17]. By computing the magnitude of Ab pressure acting on surface residues of the S protein, we predict additional positions where escape might arise in the future (S1 Data). Because we estimated here the pattern of Ab pressure on the spike in its close form, our model did not correctly predict the accessibility of residues, such as 453, which are vulnerable to Ab pressure in the S protein open configuration.

We propose here a simple geometrical interpretation of the surface mutational landscape of that spike that could inform, based on sequences and the 3D structure alone, whether a dominant component of virus evolution is evasion from Abs. This technique could serve as an indicator of the evolutionary stage in the infection trajectory of a virus and assess if it is on its way to becoming a circulating virus such as the seasonal flu.

## Materials and methods

### The geometry of immunogens and epitope choice

The first input to our model was an atomistic description of the geometry of our immunogens, which we generated from available structural information and pdb files [45,70]. For HA and S, solvent-accessible residues were identified using pymol script “findSurfaceResidues” (https://pymolwiki.org/index.php/FindSurfaceResidues), which identifies atoms with a solvent accessible area greater than or equal to 20 Ang^2^ (HA) and 15 Ang^2^ (S). We then found the residues to which those atoms belong. This selection criterion gives a uniformly distributed set of residues on the face of HA and S (see S1A and S1B Fig). A total of 228 epitopes (residues) were chosen for HA and 255 epitopes for S. Residues not defined as epitopes were either not present in the pdb files, or not identified by the pymol script.

We constructed a simplified model of the influenza virus, in which 40 HA molecules are arranged in a fixed conformation on a sphere of radius equal to 16nm (a value chosen for computational tractability). The model recapitulates [24] the average spacing between adjacent HAs on the influenza viral surface of ~ 14 nm (Harris et al., 2013 [78]). We also constructed a simplified model of the coronavirus based on the cryo-EM images of the SARS virus, in which 65 S molecules in closed form [45] are arranged in a fixed conformation on a sphere of radius equal to 87nm [46], resulting in a density of 0.27 spikes pre 100nm^2^.

Steric constraints affect the accessibility of Abs to epitopes, changing the on-rate, thus modulating the affinity. To compute the relative magnitude of this effect for different epitopes presented by immunogens with different geometries, we employed MD simulations. In these simulations, a Lennard-Jones (LJ) potential describes the interactions of an IgG Ab model with the immunogen, and a separate Morse-potential is used to model interactions of the antigen binding region of the Ab to its specific cognate epitope (see S1 Table). To estimate the steric effects alone, we used MD simulations (Lammps software) [71] to compute the average time for the Ab antigen-binding region (S1C and S1D Fig) to find the target epitope for the first time, which is called a “first passage time.” By running simulations multiple times and then averaging the results over many simulations, we estimated the mean first-passage time to the epitope. The inverse of the mean first-passage time is the on-rate. The on-rate of the first arm of the Ab model is a proxy for Ab geometry-dependent component of affinity to a residue.

### Coarse-grained model of the antibody and the immunogen

We constructed a coarse-grained model of an IgG Ab using 8 beads, and of the immunogen (see S1 Fig). In [72], a model of the Ab was suggested, built from ellipsoids and spheres. Here, we built our Ab model using spheres of different sizes to approximate the same dimension and flexibility of an IgG. The MD simulation system is composed of different beads (see S1 Table). This size of the beads was chosen such that the distance between the two Fabs is approximately 15nm and the length of the Ab arm is 7nm [73]. The size of the Fc region is chosen to be 5nm [74] (see S1 Table). To construct the 7nm arm we use 3 beads (types 4,5,6 –S1B and S1C Fig, and S1 Table), where nearest-neighbor beads are connected with rigid bonds of length 1.75nm. Bead type 4 (arm hinge) is connected to bead 3 (Fc hinge) by a rigid bond of length 1.75nm. The epitope bead (type 7, S1 Table) was chosen to have the same size as the Fab beads (1.75nm) (S1 Table). The beads along the arm (type 4,5,6) are on a straight line (no kink), and the middle bead (type 6) is larger, to approximate the elongated ellipsoid shaped arm of the Ab [72].

The average angle between the two arms of the Ab fluctuate with a mean of 120 degrees and obeys the harmonic potential

$$U(\theta )=\kappa {\left(\theta -\theta_{0}\right)}^{2},$$ (S1)

with *θ*_0_ = 0.66radian and *κ* = 10k_b_T/radian^2^, resulting in a relatively rigid model of the Ab (De Michele et al., 2016 [72]).

The system is integrated using a Langevin thermostat under “fix nve” to perform performs Brownian dynamics simulations (see https://lammps.sandia.gov/doc/fix_langevin.html).

The Fab bead interacts with the respective epitope bead via the Morse potential

$$E=D_{0}[e^{-2\alpha (r-r_{0})}-2e^{-2\alpha (r-r_{0})}]\;\mathrm{for}\;r<r_{c},$$ (S2)

where *r*_0_ = 1.75nm is the distance between the Fab bead and an epitope bead at which the LJ energy between them is zero, and the cutoff radius *r*_*c*_ = 2.2nm. *D*_0_ = 50 is the energy and the bond fluctuation scale *α* = 1nm^−1^: the Morse potential only serves to anchor the 1^st^ arm to the epitope allowing the second arm to search for a second epitope.

The beads interact with the LJ potential

$$E=4\varepsilon [{\left(\frac{\sigma_{i,j}}{r}\right)}^{12}-{\left(\frac{\sigma_{i,j}}{r}\right)}^{6}]\;\mathrm{for}\;r<r_{c},$$ (S3)

where *ε* = 1, *σ*_*i*,*j*_ is the interaction distance between beads i and j, and the cutoff radius is *r*_*c*_ = 2^1/6^*σ*_*i*,*j*_. The values of *σ*_*i*,*j*_ are detailed in S2 Table. The LJ interaction distance *σ*_*i*,*j*_ between all beads composing the Ab arm (types 4, 5, 6), and the epitope bead (type 7) is 1.75nm to construct the 7nm long arm. The LJ self-interaction distance of the Ab arm bead (type 6) was taken to be 4.2nm (S1 Table) to maintain an angle of approximately 120 degrees between the arms. The interaction distance of other pairs of beads is the sum of their radii (S2 Table).

### Estimating the on-rates to the epitopes

The on-rate to each of the residues is estimated using MD simulations. Each simulation runs for a predetermined amount of time and we find the diffusion-limited first passage time of one of the Fabs to the neighborhood of the target residue. The on-rate for the first arm to find an epitope is given by

$$\omega_{Ep}=f_{Ep}/(\frac{1}{N_{Sim}}\sum_{i}\tau_{Ep,i})$$ (S4)

where *τ*_*Ep*,*i*_ is the time estimated from simulation *i*, for the Ab to find epitope *E*_*p*_, to find epitope *E_p_*, *f_Ep_* is the fraction of simulations where the arm finds the epitope, and *N*_*Sim*_ is the number of independent simulations we perform. We performed independent MD simulations to estimate *ω*_*Ep*_ for each epitope (7 independent simulations for the HA trimer, 12 for the influenza virus model, 17 independent simulations for the S protein trimer, 9 for the coronavirus model). See S5 Fig for simulation convergence.

### Viral sequences

The sequences analyzed here of the seasonal influenza H1N1 and the 2009 influenza pandemic are from [40], coming from www.gisaid.org. For the seasonal flu (pre-pandemic influenza H1N1), 577 sequences from the years 1918–1957 and 1977–2009 were analyzed. For the pandemic flu, 431 sequences from the years 2009–2017 were analyzed. SARS-CoV-2 sequences were downloaded on June 18^th,^ 2020 from www.gisaid.org. Out of a total of 1,981,163, only high-quality (complete) sequences of length 1274 amino acid (681391 sequences) were analyzed. The consensus sequence of SARS-CoV-2 was calculated using the BLOSUM50 scoring matrix in Matlab. Sequences of the sarbecovirus subgenus were downloaded from www.ncbi.nlm.nih.gov. (see Table 1). The alignment of those sequences was done using ‘GONNET’ scoring matrix in Matlab. Find an interactive, time-dependent comparison of the mutability map to the on-rate map model here https://amitaiassaf.github.io/SpikeGeometry/SARSCoV2EvoT.html.

### Epitope clustering

As the surface features of the spike appeared to be an important factor in determining Ab on-rate, we applied a non-linear mapping (manifold learning) algorithm—diffusion maps [75] on the epitopes’ positions (S2A-i and S2B-i Fig) and used the first three components (S2A-ii and S2B-ii Fig). We then applied the k-means clustering algorithm [76] (spectral clustering) to aggregate residues in this space into epitope clusters (S2A-iii and S2B-iii Fig). To determine the optimal number of clusters *k* for comparison between the two maps, we first estimated the Total Within Sum of Squares for different cluster numbers (S4A Fig) and used the elbow method to choose *k* = 60 [77].

## Supporting information

S1 Table Dimensions of the elements constructing the coarse-grained models. Description of the elements constructing the coarse-grained antibody model (S1C and S1D Fig) and the immunogens (Figs 1A-i, 1B-i, 3A-i, 3B-i, S1A and S1B).
(DOCX)

S2 Table LJ interaction parameters. Values of *σ*_*i*,*j*_ in nm.
(DOCX)

S1 Data Ab on-rate and residue entropy for influenza and SARS-CoV-2. Summary of data presented in the paper: on-rate of the first arm Ab for both influenza and SARS-Cov-2 Spikes against epitopes (resides) for the virus and trimer presentation, the entropy of epitopes. The functional role of mutations in key residues for SARS-CoV-2.
(XLSX)

S1 Fig S and HA antigen-antibody models and antibody. **(A)** Set of 255 distinct residues on the surface of the S protein of SARS-CoV-2 were identified as epitopes. See also Materials and Methods. **(B)** Set of 228 distinct residues on the surface of HA were identified as epitopes. **(C-D)** Schematic representation of the antibody (Ab). The large blue bead represents the Fc part of the Ab. The two magenta beads are the arms, and the yellow beads are the Fab section of the arms. The model also contains hinge beads between the Fc and the arms. For full description see “Coarse-grained model of the antibody”).
(TIF)

S2 Fig Clustering of residue to epitope clusters. **(A-B)** Panels i. HA protein (A) and the S protein (B). Each circle corresponds to a surface residue (epitope) and was colored differently for illustration. Panels ii. 2d projections of the first four eigenvectors of the epitope positions following diffusion map decompositions. Panels iii. Clustering of the surface residues of the spike protein using k-means clustering algorithm applied to the spectral decomposition shown in panel ii (*k* = 60).
(TIF)

S3 Fig Sarbecovirus subgenus phylogenetic tree. The sequence origin is detailed in Table 1.
(TIF)

S4 Fig Clustering of spike surface residues (epitopes). **(A)** The Total Within Sum of Squares as a function of the number of clusters computed for seasonal influenza spike HA. Related to Fig 2 **(B)** The Total Within Sum of Squares as a function of the number of clusters computed for the sarbecovirus subgenus spike. Related to Fig 3.
(TIF)

S5 Fig MD simulations convergence. Histogram showing the fraction of simulations that finished with a successful binding event. For each immunogen we show the number of epitopes for which a certain fraction of the simulation ended in a successful binding event of a single-arm: (A) HA trimer; (B) influenza virus; (C) S protein timer; (D) SARS-CoV-2 Virus.
(TIF)
