# Supplementary material for: Viral surface geometry shapes influenza and coronavirus spike evolution through antibody pressure
Source: PLoS Comput Biol. 2021 Dec 13;17(12):e1009664. doi: 10.1371/journal.pcbi.1009664 (PMC8699686; doi:10.1371/journal.pcbi.1009664)
Supplement: S1 Table — Description of the elements constructing the coarse-grained antibody model (S1C and S1D Fig) and the immunogens (Figs 1A-i, 1B-i, 3A-i, 3B-i, S1A and S1B). (DOCX) [file pcbi.1009664.s001.docx]

| Index | Description | Size $\sigma_{i,i}$ | Bead color in Figure S1 |
| --- | --- | --- | --- |
| 1 | “Structural” atom of the immunogen. Not an epitope. | 0.8nm | - |
| 2 | Fc of the Ab | 5nm | Blue |
| 3 | Hinge bead connecting the Fc bead to the two arms hinges | 1nm | Orange |
| 4 | Hinge beads in the Ab arms | 1.75nm | White |
| 5 | Fab | 1.75nm | Yellow |
| 6 | Arm bead in the Ab | 4.2nm | Magenta |
| 7 | Epitope beads | 1.75nm | - |

S1 Table **Dimensions of the elements constructing the coarse-grained models.** Description of the elements constructing the coarse-grained antibody model (S1C and S1D Figs) and the immunogens (Figs 1A-i, 1B-i, 3A-i, 3B-i, S1A, and S1B).
