# Supplementary material for: Viral surface geometry shapes influenza and coronavirus spike evolution through antibody pressure
Source: PLoS Comput Biol. 2021 Dec 13;17(12):e1009664. doi: 10.1371/journal.pcbi.1009664 (PMC8699686; doi:10.1371/journal.pcbi.1009664)
Supplement: S2 Table — Values of σi,j in nm. (DOCX) [file pcbi.1009664.s002.docx]

| Bead index | 1 | 2 | 3 | 4 | 5 | 6 | 7 |
| --- | --- | --- | --- | --- | --- | --- | --- |
| 1 | - | 1.275 | 1.275 | 1.275 | 1.275 | 1.275 | 1.275 |
| 2 |  | - | 3. 375 | 3. 375 | 3.375 | 3. 375 | 1.75 |
| 3 |  |  | - | 0.50 | 1.75 | 1.75 | 1.75 |
| 4 |  |  |  | - | 1.75 | 1.75 | 1.75 |
| 5 |  |  |  |  | - | 1.75 | 1.75 |
| 6 |  |  |  |  |  | - | 1.75 |
| 7 |  |  |  |  |  |  | - |

S2 Table **LJ interaction parameters.** Values of $\sigma_{i,j}$ in nm.
