# Supplementary figures and images for: Viral surface geometry shapes influenza and coronavirus spike evolution through antibody pressure
Source: PLoS Comput Biol. 2021 Dec 13;17(12):e1009664. doi: 10.1371/journal.pcbi.1009664 (PMC8699686; doi:10.1371/journal.pcbi.1009664)

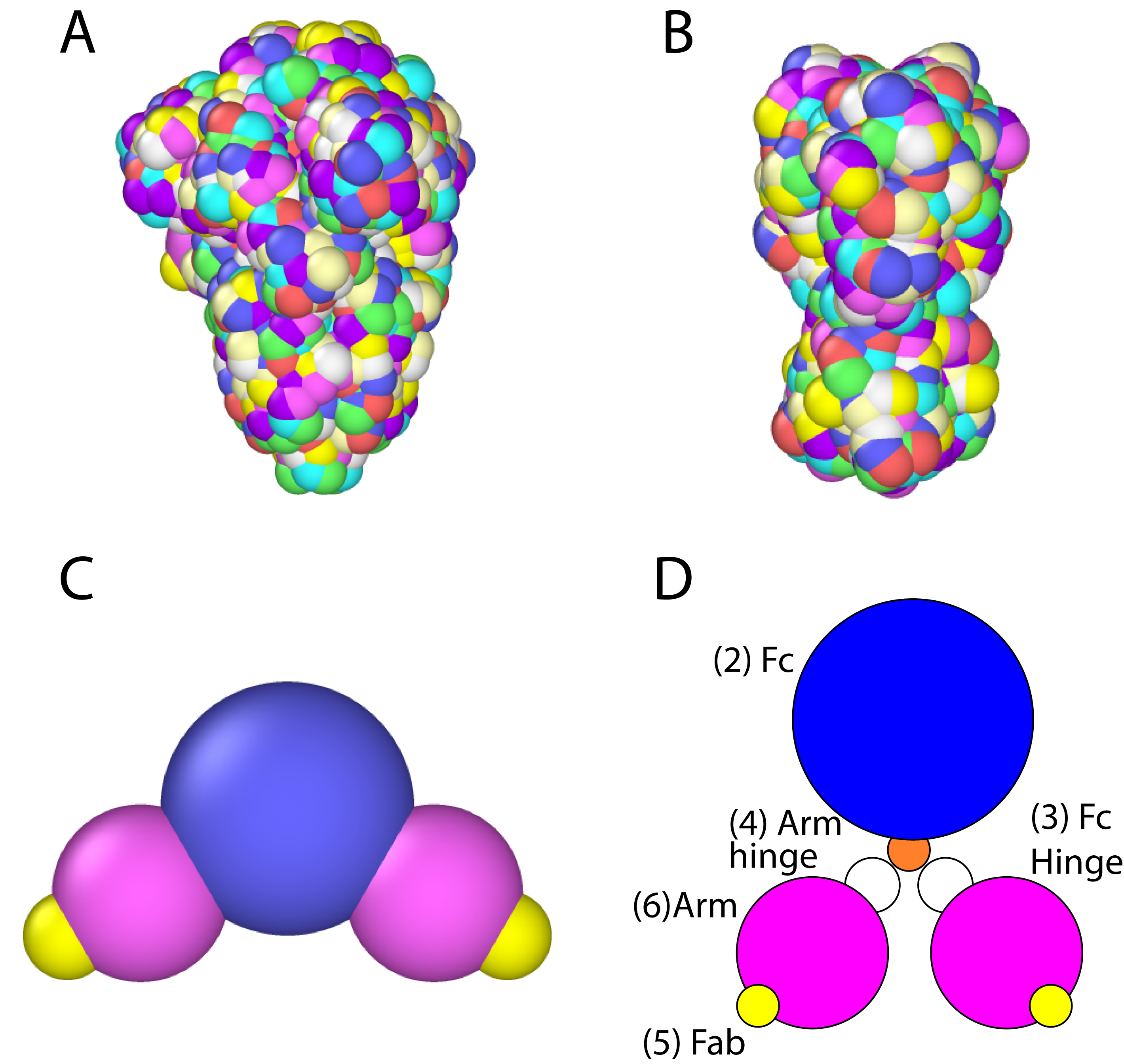

Supplement: S1 Fig — (A) Set of 255 distinct residues on the surface of the S protein of SARS-CoV-2 were identified as epitopes. See also Materials and Methods. (B) Set of 228 distinct residues on the surface of HA were identified as epitopes. (C-D) Schematic representation of the antibody (Ab). The large blue bead represents the Fc part of the Ab. The two magenta beads are the arms, and the yellow beads are the Fab section of the arms. The model also contains hinge beads between the Fc and the arms. For full description see “Coarse-grained model of the antibody”). (TIF) [file pcbi.1009664.s004.tif]

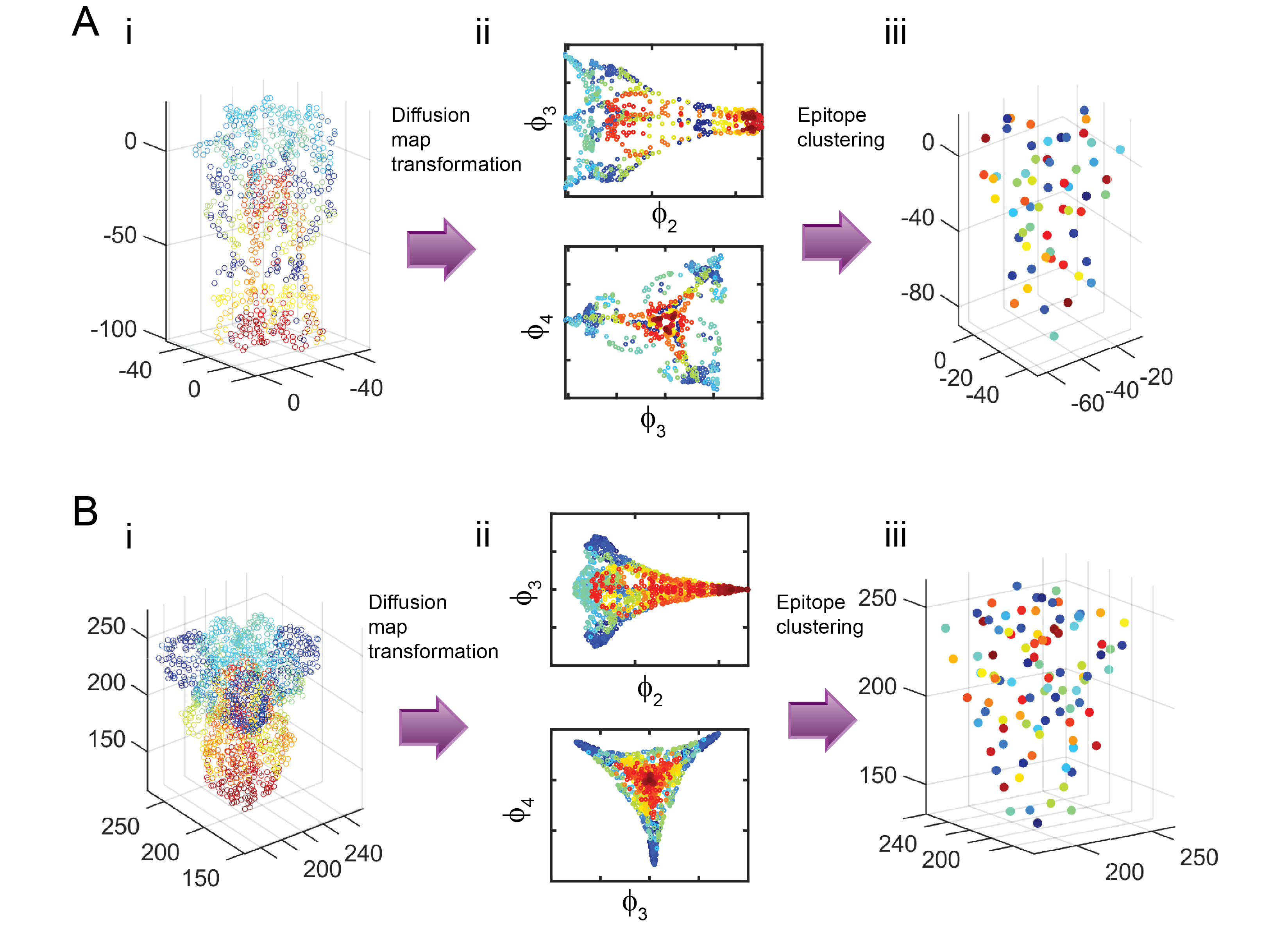

Supplement: S2 Fig — (A-B) Panels i. HA protein (A) and the S protein (B). Each circle corresponds to a surface residue (epitope) and was colored differently for illustration. Panels ii. 2d projections of the first four eigenvectors of the epitope positions following diffusion map decompositions. Panels iii. Clustering of the surface residues of the spike protein using k-means clustering algorithm applied to the spectral decomposition shown in panel ii (k = 60). (TIF) [file pcbi.1009664.s005.tif]

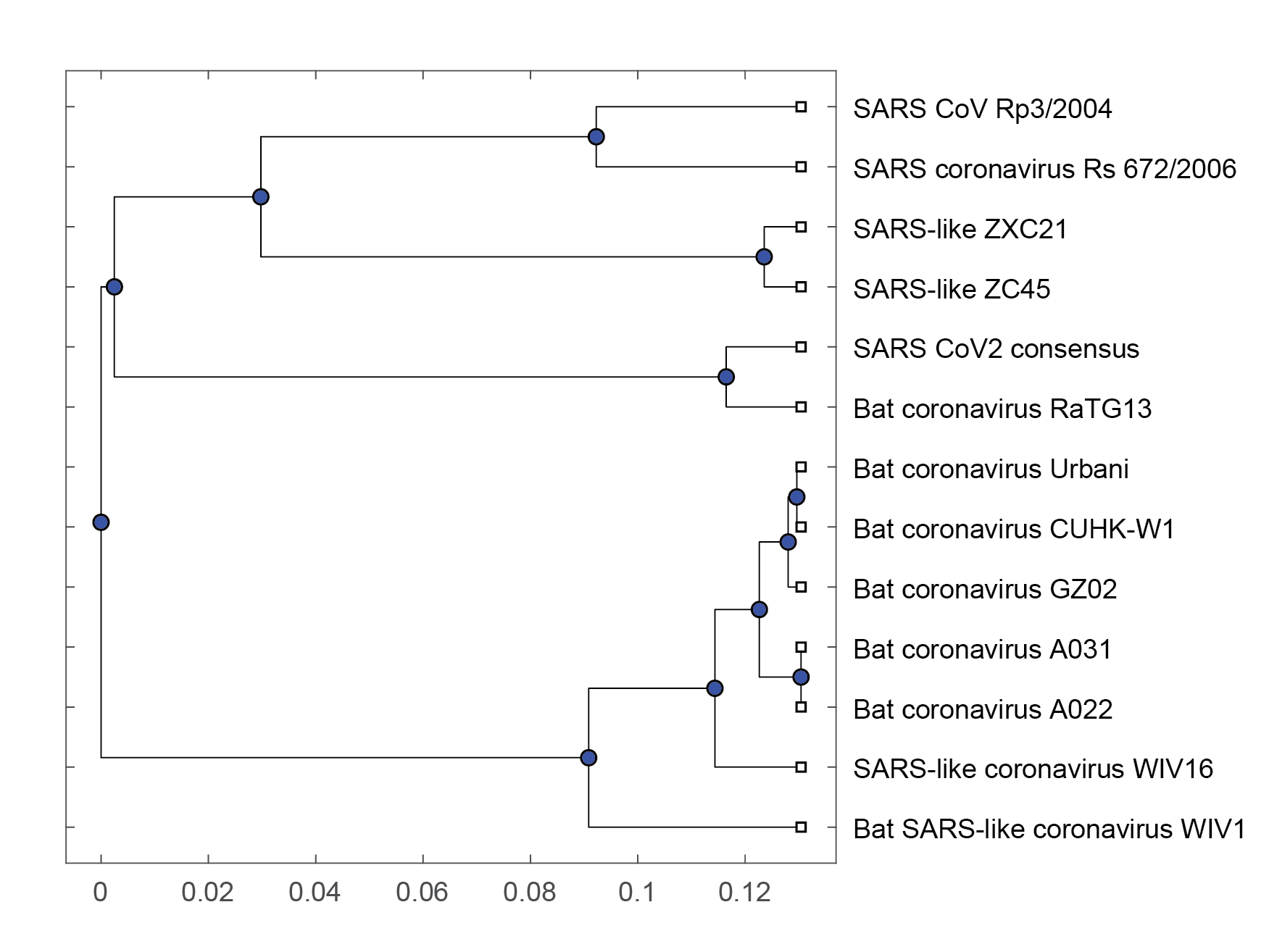

Supplement: S3 Fig — The sequence origin is detailed in Table 1. (TIF) [file pcbi.1009664.s006.tif]

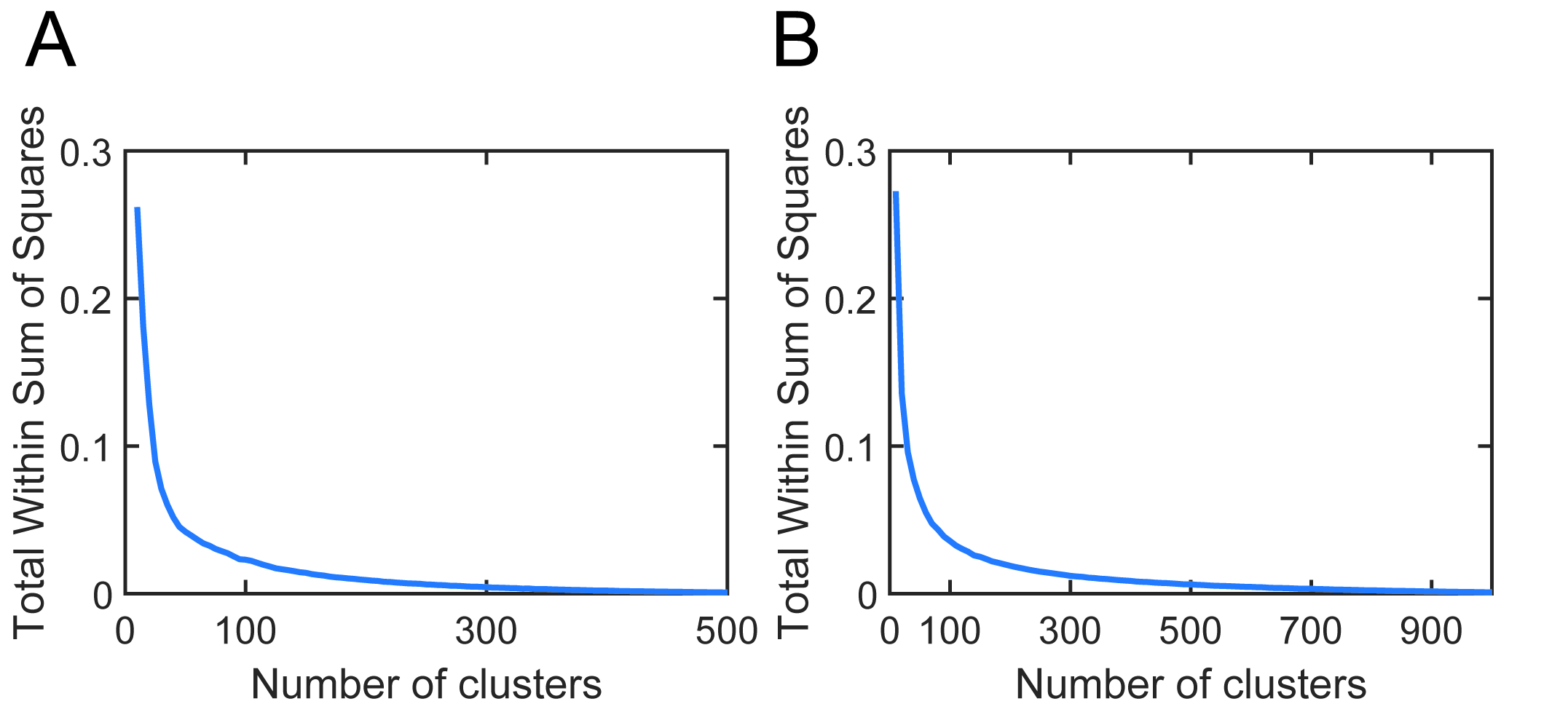

Supplement: S4 Fig — (A) The Total Within Sum of Squares as a function of the number of clusters computed for seasonal influenza spike HA. Related to Fig 2 (B) The Total Within Sum of Squares as a function of the number of clusters computed for the sarbecovirus subgenus spike. Related to Fig 3. (TIF) [file pcbi.1009664.s007.tif]

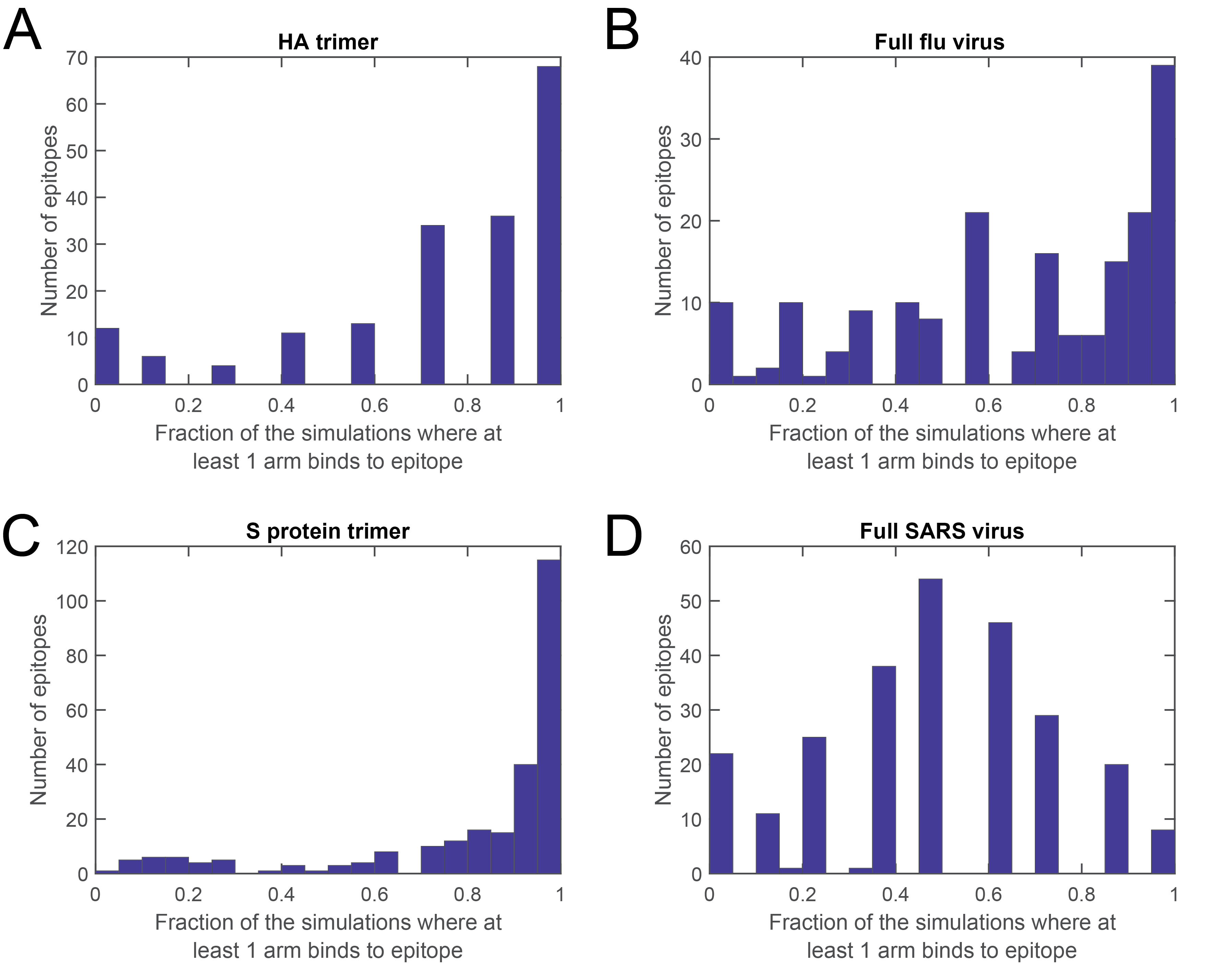

Supplement: S5 Fig — Histogram showing the fraction of simulations that finished with a successful binding event. For each immunogen we show the number of epitopes for which a certain fraction of the simulation ended in a successful binding event of a single-arm: (A) HA trimer; (B) influenza virus; (C) S protein timer; (D) SARS-CoV-2 Virus. (TIF) [file pcbi.1009664.s008.tif]
